# Efficacy of Bacterial Nanocellulose in Hard Tissue Regeneration: A Review

**DOI:** 10.3390/ma14174777

**Published:** 2021-08-24

**Authors:** Anuj Kumar, Sung-Soo Han

**Affiliations:** 1 School of Chemical Engineering, Yeungnam University, 280 Daehak-ro, Gyeongsan 38541, Korea; 2 Institute of Cell Culture, Yeungnam University, 280 Daehak-ro, Gyeongsan 38541, Korea

**Keywords:** bacterial cellulose, nanocellulose, bone tissue regeneration, additive manufacturing, natural polymers, biodegradation, toxicity and cellular response

## Abstract

Bacterial nanocellulose (BNC, as exopolysaccharide) synthesized by some specific bacteria strains is a fascinating biopolymer composed of the three-dimensional pure cellulosic nanofibrous matrix without containing lignin, hemicellulose, pectin, and other impurities as in plant-based cellulose. Due to its excellent biocompatibility (in vitro and in vivo), high water-holding capacity, flexibility, high mechanical properties, and a large number of hydroxyl groups that are most similar characteristics of native tissues, BNC has shown great potential in tissue engineering applications. This review focuses on and discusses the efficacy of BNC- or BNC-based biomaterials for hard tissue regeneration. In this review, we provide brief information on the key aspects of synthesis and properties of BNC, including solubility, biodegradability, thermal stability, antimicrobial ability, toxicity, and cellular response. Further, modification approaches are discussed briefly to improve the properties of BNC or BNC-based structures. In addition, various biomaterials by using BNC (as sacrificial template or matrix) or BNC in conjugation with polymers and/or fillers are reviewed and discussed for dental and bone tissue engineering applications. Moreover, the conclusion with perspective for future research directions of using BNC for hard tissue regeneration is briefly discussed.

## 1. Introduction

Hard tissue (bone or dental) regeneration has been a great challenge in surgical procedures due to traumatic injuries, musculoskeletal pathologies (e.g., low back pain, fractures, congenital defects, bone infection or tumors, scoliosis, and osteoporosis), maxillofacial pathologies, and other diseases such as osteomyelitis, osteitis, and osteoarthritis [1,2,3]. However, the dynamic and complex bone structure exhibits outstanding tissue regenerative characteristics [4], but the poor healing ability of large and complex defects in bone caused by above mentioned orthopedic reasons presents a major challenge clinically. Therefore, these defects need alternative procedures to regenerate appropriate hard tissues [2]. Owing to various imitations of conventional procedures (e.g., autografts, allografts, xenografts, or artificial metal implants), natural and/or synthetic polymer-based scaffolds have shown a great potential for hard tissue regeneration process due to their suitable moldability, bioinertness, excellent biocompatibility, ease of manipulation in degradation and mechanical properties to mimic three-dimensional (3D) architecture of extracellular matrix (ECM) of native hard tissues [2].

Various natural polymers have been used in tissue engineering areas, such as collagen, gelatin, hyaluronic acid, chitosan, and cellulose due to their most similar characteristics of native tissues [5]. Here, cellulose as the most abundant biopolymer is highly desired in biomedical areas due to its renewable nature, lost price, and in vitro biodegradability. Cellulose can be produced by plants, fungi, algae, and bacteria [6,7]. Therefore, natural polymers, specifically polysaccharides-based bioactive composites, are applied more promisingly in tissue engineering, especially in hard tissue regeneration. Nanocelluloses-based 3D scaffolds are more advantageous over other nanomaterials while fabricating composite scaffolds for hard tissue engineering due to their renewable nature, high specific surface area and aspect ratio, excellent biocompatibility, and nano-mechanical properties [8].

Unlike other nanocelluloses (e.g., cellulose nanocrystals or nanofibres), bacterial nanocellulose (BNC) exhibits unique properties such as chemically pure nanofibrous network, high water-holding capacity, excellent biocompatibility, and high mechanical properties that make it highly suitable for the hard tissue regeneration process. This nanofibrous network of BNC resembling native ECM exhibits remarkable effects on cell adhesion and proliferation and is highly desired for making tissue scaffolds [9]. Therefore, the main purpose of this review is to provide an overview of the synthesis and characteristics of BNC to gain a better understanding of functional properties and its efficacy in hard tissue engineering applications. Here, the most recent advances of BNC or BNC-based biomaterials in hard tissue engineering are recapitulated and discussed to build up knowledge for future research directions. In this review, first, we describe the efficacy of nanocellulose in tissue engineering and then a precise description of the synthesis of BNC and its properties, including solubility, biodegradation ability, thermal stability, toxicity and cellular response, antimicrobial activity, and further desired modification methods to enhance properties of BNC or BNC-based biomaterials. In addition, the efficacy of BNC or BNC-based scaffolds for hard tissue regeneration is reviewed and discussed. Finally, the conclusion and future perspective are provided for possible future research directions.

## 2. Nanocellulose in Tissue Engineering

**Figure 1:**
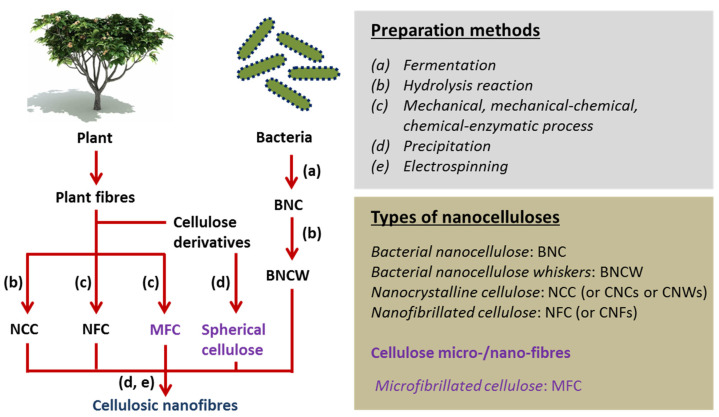
Schematic representation of the sources, preparation methods, and classification of nanocelluloses. Adapted from [14].

Nanocellulose-based biomaterials with various forms have promising uses in tissue engineering areas due to their excellent biocompatibility, water absorption and retention, optical transparency, and chemo-mechanical characteristics [10,11,12]. Here, nanocellulose refers to the cellulose with different crystalline content and shape/size at least one dimension in nanoscale; for example, these are categorized mainly as bacterial nanocellulose (BNC) produced by using specific bacteria strains or BNC whiskers (as hydrolysate of BNC), whereas nanofibrillated cellulose (NFC or nanofibres (CNFs)) and nanocrystalline cellulose (NCC: nanocrystals (CNCs) or nanowhiskers (CNWs)) produced or extracted by various methods as chemical or mechanical or enzymatic or their combinations [13,14]. A schematic of the sources, extraction strategies, and classification of nanocelluloses are shown in Figure 1 [14].

High stiffness and crystallinity of CNCs with effective surface charge due to acid-hydrolysis exhibited suitable prospects for biomedical applications. Depending upon used treatment methods, CNCs have a typical diameter in the range of 5–50 nm and length in the range of 100–300 nm or several micrometers as derived from tunicates, crystallinity in the range of 50–90%, and elastic moduli as ~105–168 GPa. Compared to CNCs, NFC has a diameter ranging 10–100 nm and a length over 10 μm based on source and methods. NFC has soft and flexible fibrils and low crystallinity as compared to CNCs and BNC. In addition, tensile Young’s modulus (~30 GPa) of NFC is greatly lower than that of CNCs and can be varied based on the used method (e.g., delamination). Both CNCs and NFC exhibit shear-thinning behaviors. In comparison with CNCs and NFC, BNC is synthesized generally as continuous fibers having a diameter of 10–100 nm with high crystallinity of 74–96% and Young’s modulus of 78–114 GPa that is comparable with CNCs, but higher than that of NFC. However, the mechanical properties of BNC pellicles were also measured in the order of 10 GPa [13,15]. Therefore, these forms of nanocelluloses have been widely applied to prepare foams and gels (including aerogels, cryogels, and xerogels) by using various green and cost-effective methods for controlling structural features and biological characteristics, including scaffolds for various tissue engineering applications [8].

In parallel to other nanocelluloses, recently, BNC or BNC whiskers (BNCW) have shown great attention in tissue engineering areas, particularly for bone tissue regeneration. BNC, as exo-polysaccharide (β-D-glucopyranose), has been synthesized by using particular bacteria strains. The molecular structure of BNC is the same as plant-based nanocelluloses, but some physical and chemical characteristics such as high purity and crystallinity, nanofibrous network, excellent mechanical properties, high (approximately 99%) water-retention ability within the network are different [16]. In addition, BNC is free of lignin, hemicelluloses, pectin, and other elements as compared with plant-based cellulose [17]. Therefore, its water-holding capacity and nanofibrous morphology (similar to natural ECM protein, i.e., collagen [18]) show the potential efficacy of BNC for cellular immobilization and compatible responses. However, the feasible application of BNC is quietly dependent on its production cost. The production of BNC requires high capital investment to improve the operational capacity. Therefore, the commercial use of BNC at low cost is a major challenge in biomedical areas, and advanced bioprocess technologies are highly desired rather than conventional fermentation methods [19,20]. Considering this issue, comprehensive research for optimizing factors and parameters for large production at low or optimum cost is under investigation [21,22,23,24]. In the next section, the synthesis and properties of BNC and their fabrication methods for tissue engineering are precisely reviewed, including their advantages and disadvantages.

## 3. Bacterial Nanocellulose

### 3.1. Synthesis

**Figure 2:**
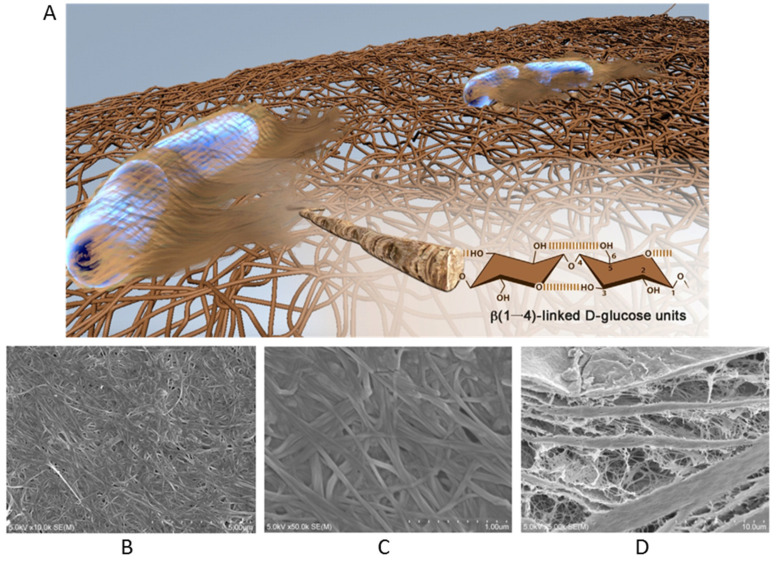
(**A**) A representative model image of a 3D nanofibrous network of BNC as secreted by *A. xylinus* bacteria (including detailed hydroxyl functional groups of the highlighted single nanofibril). Reproduced with permission from [29]. Copyright 2016 Elsevier. SEM images of BNC surface at different magnifications as (**B**) 10.0 kx and (**C**) 50.0 kx. (**D**) cross-section of BNC (5.0 kx). Reproduced from [28].

BNC is an exopolysaccharide and has the analogous chemical structure as plant-based celluloses (β-1,4-linked anhydro-D-glucose units) and possesses high intermolecular hydrogen bonds among hydroxyl groups, which make it insoluble in water and thereby turning into non-resorbable and bio-stable biomaterial (inside the human body) [17]. BNC was first synthesized using bacteria (*Kamagataeibacter xylinus*) in 1886 by A. J. Brown [25] through a cellulose synthase enzyme, where the activity of the enzyme is dependent on Mg^2+^ ions with optimal pH between 7.5 and 8.5 at 30 °C. Generally, BNC is synthesized by using various bacterial genera (e.g., *Aerobacter*, *Achromobacter*, *Acetobacter*, *Azotobacter*, *Enterobacter*, *Agrobacterium*, *Sarcina*, *Bacillus*, *Escherichia*, *Rhizobium*, *Klebsiella*, *Salmonella* are very well known to produce cellulose) [20,26]. Most commonly, *Gluconacetobacter xylinus* (a Gram-negative bacteria, also known as *Acetobacter xylinus*) has been used to produce BNC with a chemical structure identical to plant cellulose (as molecularly (C_6_H_10_O_5_)_n_ with β(1,4)-D-glucose repeating units). A single cell of G. xylinus may polymerize up to 200 × 10^3^ glucose units assembled in a ribbon-like bundle of twisted nanofibrils extruded into the surrounding culture medium, even during cell division for elongation along cell envelop [27]. The obtained BNC can have nanofibrils of 1–25 nm wide and 1–9 μm in length as assigned to 2–18 × 10^3^ glucose units [16,27]. For example, the surface and cross-section morphology of BNC can be shown in Figure 2, which constitutes a laminated structure containing porous interconnecting layers and densely compact layers. In addition, cellulosic nanofibrils exhibit uniform ‘web-like’ structures on the BNC surface [28].

In static culture, these ribbon-like fibrils as produced by bacteria accumulate at liquid/air interface to form a surface mat, termed as BNC pellicle. The use of *G. xylinus* as a model organism to produce BNC as pure ECM with metabolic inertness is advantageous over the production of plant-based nanocellulose [20]. The supramolecular structure of BNC and its physical and mechanical properties are directly dependent on the used production methods [20,30]. Therefore, BNC can be obtained by using static or agitated fermentation methods, where static culture facilitates uniaxial-oriented ribbons of cellulose, while agitated culture formed a disordered, curved, and overlapped ribbon-like morphology of cellulose [17,31]. However, these two methods also show some disadvantages; for example, static culture generally produces low yield with a long duration of culture and used manpower, whereas agitated culture switches bacteria used to produce BNC toward cellulose negative mutants due to the overgrowth of bacteria in the culture environment, thereby decreased the production [32,33].

In the last two decades, various production strategies for BNC have been applied, but commercial production is still dependent on the conventional method (i.e., static culture in shallow vessels with or without minimal biotechnological procedures) [30]. To overcome the demerits of static and agitated culture methods, currently, the use of bioreactors demonstrated an ability to facilitate suitable productivity of BNC under directed experimental parameters [20,34], including substrate-based approaches to supplement the production of BNC [20]. Although bioreactor culture has enhanced the production yield of BNC to some extent, a high yield of BNC with suitable properties by the bioreactor method is not efficiently achieved yet. Therefore, static culture is still the most common method to produce BNC with suitable quality, even though the culture time is long and production is low. Moreover, the strategies of BNC production can be categorized into three ways: (1) static culture, (2) agitated culture, and (3) bioreactor culture [35,36]. High cost and low productivity are two major challenges in producing BNC. Therefore, the current research is focused on using high-yield mutants developed by genetic techniques or optimized culture conditions for enhancing the production yield of BNC at a low cost [37]. In this case, one of the important factors in the production of BNC is the modification of microarchitecture of BNC as a result of culture medium conditions, for example, using ethanol [38,39], coconut water [40], agar [21], polymers (e.g., carboxymethyl cellulose [41], sodium alginate [42]), composite supports (of polyolefin and plant materials) [20,32], sweet-lime pulp waste [43], paraffin-based wax [44], etc. Apart from these advancements, the high production yield of BNC with a lost cost is still a challenge.

### 3.2. Properties

BNC has more remarkable characteristics such as highly pure ECM without lignin, hemicellulose, pectin, and other impurities as compared to plant-based cellulose. In addition, BNC possesses high crystallinity up to 95% as well as excellent mechanical properties (Young’s modulus of the monofilament up to 114 GPa) [45]. In addition, considering the complexity of the mechanical loading conditions in the physiological environment (human body), it is investigated that BNC hydrogel exhibits non-linear time-dependent rheological behavior (viscoelastic behavior) under uniaxial applied stress and stiffening effect induced by fiber orientation under axial stretching [46]. Therefore, owing to its hygroscopic characteristic, water-holding capacity (%), flexibility and advantageous permeability, controllable biodegradability (during synthesis) make it most beneficial for tissue engineering applications [35].

#### 3.2.1. Solubility, Biodegradation, and Thermal Stability

BNC is insoluble in aqueous media (i.e., water) and some other solvents due to its intra-molecular bonding and extensive hydrogen bonding among cellulose polymeric chains. In addition, BNC has superior thermal stability, which limits its melt-extrusion-based processing [47]. However, BNC can be processed by dissolving it into ionic liquid as solvents and then using fabrication methods, such as solvent-casting, molding, and electrospinning [47]. Further, BNC shows non-biodegradability in vivo by enzymes because the body does not produce cellulase enzymes to facilitate its biodegradation. However, the biodegradability of BNC can be modulated by using various chemical treatments or the incorporation of molecules [48,49]. The wide use of BNC in biomedical science involves the variation in different properties in terms of biodegradation and thermal stability. Here, there is considered four main factors (e.g., molecular weight, crystallinity, hydrophilicity, and modification approach) that could affect the biodegradation of BNC-based biomaterials under physiological conditions. In addition, four main hypothetical mechanisms of BNC biodegradation (e.g., hydrolysis (reaction with water molecules in tissues), oxidation (due to oxidants produced by tissues), enzymatic and physical (due to water swelling, mechanical loading, and wearing) mechanisms) have been identified in terms of in vivo degradation of BNC. In addition, pure BNC can degrade thermally as low as 190 °C and could be improved to 580 °C by modifying with an inorganic nanoscaled particle. Therefore, BNC can be made stable or degradable for the desired application by manipulating key factors and made thermally stable at higher temperatures by incorporating reinforcing agents [49].

#### 3.2.2. Antimicrobial Ability

BNC does not show the intrinsic antimicrobial property, and therefore, this important characteristic can be introduced by adding external antimicrobial agents (e.g., biopolymers or natural agents). The antimicrobial property can also be imparted within BNC films by exposing them to antibiotics (e.g., fusidic acid, levofloxacin, benzalkonium chloride) [20,50,51]. In brief, various materials such as antioxidants (e.g., propolis [52]), natural polymers (e.g., chitosan [53]), clay (e.g., montmorillonite (MMT) [54]) and antibacterial/antimicrobial agents (e.g., bio-extracts [55], silver nanoparticles (AgNPs) [56,57], copper nanoparticles (CuNPs) [58], titanium oxide (TiO_2_) [59], benzalkonium chloride [51]) can be introduced into BNC matrix to impart antibacterial or antimicrobial properties for various tissue engineering applications. For example, a titanium-aluminum-niobium (Ti6Al7Nb) bone scaffold has been manufactured by using selective laser melting and then coated in situ by BNC layer by immersion method for 7 days. Subsequently, BNC coating layer was assimilated with antibiotic (i.e., gentamycin). Here, the release of gentamycin from BNC-coated implant prevented the growth of *S. aureus* (in vitro) and confirmed the ability of the BNC-coated implant to prevent hostile microbial colonization in orthopedic applications and reduce the risk of musculoskeletal infections [60]. In another example, BNC-assisted synthesized glass-ceramic scaffolds with bioactive characteristics for hard tissue engineering have been improved with mechanical and antibacterial properties by using semiconducting oxides (e.g., TiO_2_) [61].

Moreover, the global pandemic situation due to COVID-19 has given rise to awareness to ensure the best implementation to avoid the spreading of microorganisms. The increase in infections caused by bacteria and viruses, for example, the virus SARS-CoV-2, compelled worldwide to manufacture antiviral, antioxidant, and antimicrobial materials to avoid infectious diseases that threaten public health. Although extensive research reports are available on antibacterial/antimicrobial materials, very little data are available on antiviral materials. Therefore, the combination of antibacterial/antimicrobial and antiviral chemical entities constitutes a potentially path-breaking involvement in alleviating the spreading of disease-causing agents [62]. Further, there are several antivirals that are already available, but their poor bioavailability, poor solubility, low permeability, non-targeted release, adverse side-effects, and antiviral resistance limit their applicability in potential biomedical areas [63]. However, BNC-based materials assimilated with a substance that contains significant antiviral, antifungal, anti-inflammatory, antioxidant, and antibacterial characteristics have presented great attention as biomaterials [52].

#### 3.2.3. Toxicity and Cellular Response

**Figure 3:**
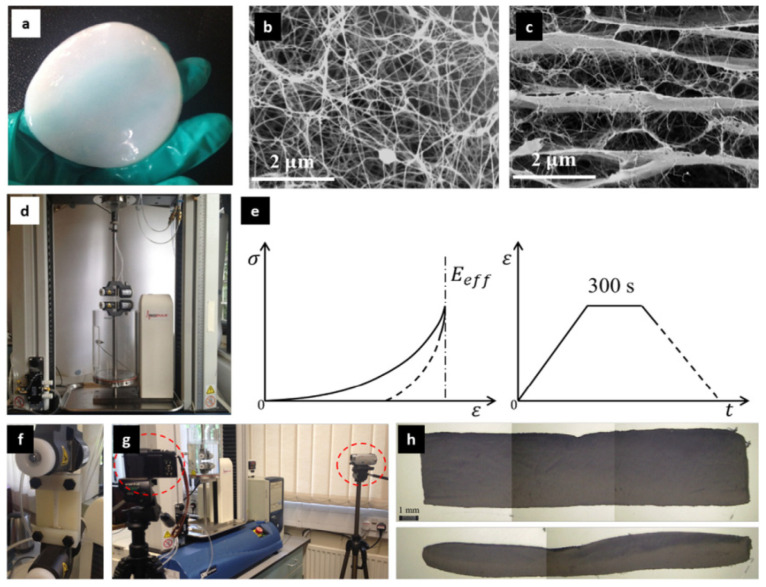
BNC hydrogel (**a**), random distribution of BNC nanofibres in fibrous layer (**b**), multi-layer network with a few crosslinks (**c**), Instron (3366) with a bath-system (BioPuls) for uniaxial in aqua tension (**d**), schematic of getting effective elastic modulus of bulk hydrogel (**e**), custom-made attachment to fix stretched specimens to prevent elastic recovery (**f**), digital cameras (two) to evaluate changes in the geometry of specimens (**g**), and optical microscopical images of freeze-dried specimens to measure total volume (**h**). Reproduced with permission from [71]. Copyright 2017 Elsevier.

The biocompatibility and hemocompatibility of BNC have been evaluated in vitro and in vivo studies [29]. BNC shows a highly porous nanofibrous network with a large specific area and outstanding biocompatibility without any inflammatory reaction or rejection [64]. Therefore, BNC is non-toxic and non-immunogenic. In vivo biocompatibility of BNC has been evaluated in detail by Helenius et al. 2006, where BNC was subcutaneously implanted in rats for 1, 4, and 12 weeks and it was found that implanted BC retained its shape without any microscopic signs of inflammation [65]. As evaluated in various studies on mice and rats, BNC is not genotoxic (rat), showed no reproductive toxicity (mice), and induced no embryotoxicity and teratogenicity effects (rats). In addition, primary eye and dermal studies (rabbits) exhibited a non-irritating effect of BNC as well as suitable biocompatibility after the subcutaneous implantation in animal models for various time durations, where BNC demonstrated no harsh inflammatory response [66]. In addition, mechanical properties of extracellular conditions or underlying substrate play an important role in guiding cell behaviors (adhesion, proliferation, and differentiation). In this case, substrate stiffness is a component that affects the interactions between substrate and cells (particularly in vitro studies) and thereby mimicking native ECM to promote cell differentiation into the right cell-lineage [67,68,69]. BNC possesses high mechanical properties (e.g., high tensile strength) and can be varied using various drying methods for different applications. For example, more outspread morphology and significant proliferation of cells on free-dried BNC were observed in 7 days, but not on air-dried BNC. Further, in 3 weeks of cell culture, no noticeable differentiation was observed for rMSCs on both types of BNCs (i.e., freeze-dried and air-dried) without using differentiation agents. Furthermore, chondrogenic differentiation was observed in some areas of the rehydrated freeze-dried BNCs, whereas osteogenic differentiation was observed on the stiffer rehydrated air-dried BNCs. Moreover, air-dried BNC showed a modulus similar to that of bone tissue, and freeze-dried BNC exhibited a modulus to that of muscle [70], as micro-/nanofibrous architecture has a promising effect on cellular behavior. Therefore, Gao et al. 2017 assessed the stiffness of nanofibres in BNC hydrogels by using the numerical-experimental framework (in aqua mechanical testing, microstructural analysis, and finite-element modeling) and showed the magnitudes between 53.7 and 64.9 GPa by calibrating modeling results with original experimental data (see Figure 3) [71]. This understanding of the mechanical response of nanofibrous BNC hydrogel is highly desirable for tissue engineering applications, specifically for hard tissue regeneration.

## 4. Surface Modification of Bacterial Nanocellulose

The suitable interfacial properties of the biomaterial facilitate suitable specific protein absorption and thereby subsequent cellular interactions (e.g., cell adhesion, proliferation, and differentiation). Therefore, the initial interaction between BNC and cells is an important factor for further cell growth and differentiation, and thereby tissue ingrowth. In general, the surface of BNC shows poor cell adhesion or attachment due to its biochemical inertness [29]. To extend the application of BNC in tissue engineering, various approaches have been applied to modify the surface of BNC to improve physical and chemical characteristics for cellular fate within the network. These modifications can be introduced by the in situ process (during cell culture) or ex-situ process (of existed BNC nanofibres) [72].

Different synthetic polymers (e.g., poly (vinyl alcohol) [73], carboxymethyl cellulose) [74], natural polymers (e.g., gelatin [75,76], alginate [77,78]), nanomaterials (e.g., hydroxyapatite (HAp) [79,80], bioactive glass (BG) [81,82], carbon nanotubes (CNTs) [83], graphene oxide (GO) [84]), proteins (e.g., collagen [85]), amino acid sequences (e.g., RGD [86]), biomolecules (e.g., growth factors [87]), antifungals (propolis [52]), antioxidants (e.g., propolis [52], fisetin [88]), anti-inflammatory (propolis [52]), and antimicrobial agents (e.g., AgNPs, TiO_2_) can be integrated with BNC by various strategies, using coating, gas plasma (e.g., nitrogen, oxygen) or irradiation (e.g., gamma) treatments, and surface sulfation or phosphorylation or other physical/chemical treatments to make BNC or BNC-based biomaterials more active as per desired applications [20]. Here, plasma techniques are effective strategies to change the BNC surface and optimize the biofunctionality without affecting native features [89].

## 5. Application in Hard Tissue Regeneration

The use of BNC-based porous materials with excellent mechanical and bioactive properties is highly desired for tissue engineering applications to support and maintain cell proliferation and differentiation for appropriate tissue ingrowth. Apparently, the first report on the use of BNC in the biomedical area was the development of BioFill as a wound dressing in 1990 (Produtos Biotechnologicos, Curitiba, PR Brazil) for treating severe burns, skin grafting of wounds, chronic skin ulcers [90], and later in 2001 on artificial blood vessel based on BNC for microsurgery [91]. After these inventions, extensive research studies have been reported for various biomedical applications, including tissue engineering. BNC is comprising of a nanofibrous network and containing promising physicochemical and biological properties, including the similarity of its fiber with the collagenous fiber of bone [92]. In this section, the efficacy of BNC or BNC-based biomaterials is reviewed and discussed by focusing on hard tissue regeneration.

### 5.1. Bacterial Nanocellulose (as Sacrificial Template)

**Figure 4:**
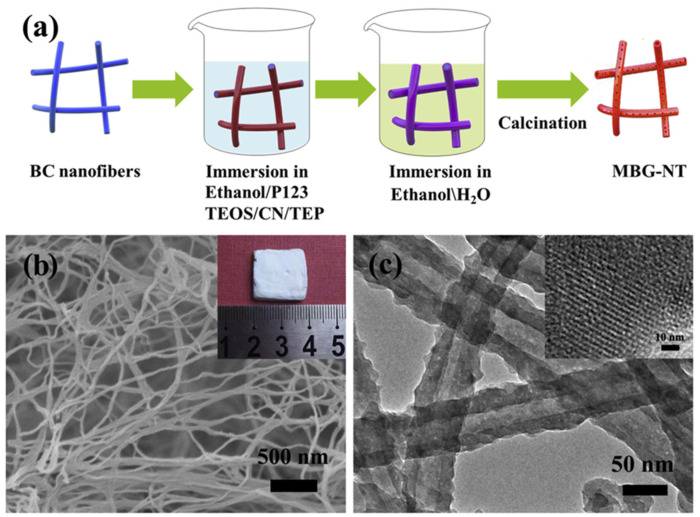
Schematic of the fabrication method of MBG-NT scaffold (**a**), SEM (**b**), and TEM (**c**) images of MBG-NT scaffold with a digital image (inset) and HRTEM image (inset), respectively. Reproduced with permission from [82]. Copyright 2019 Elsevier.

Various 3D porous scaffolds with interconnected pore-network have been developed by using BNC as a sacrificial template. For example, Luo et al. 2016 prepared a 3D nanofibrous BG scaffold by using BNC as a sacrificial template followed by calcination (600 °C, 4 h). As-developed 3D BG nanofibrous scaffolds showed an interconnected porous network with a 16 nm diameter of a nanofibre. In addition, the scaffold exhibited high bioactivity [93]. Wen et al. 2018 used amino-modified BNC as a sacrificial template for preparing 3D nanofibrous BG (NBG) scaffolds by using a modified sol-gel strategy under ultrasonic treatment followed by calcination (700 °C, 3 h) for bone tissue regeneration. In this study, amino-modified BNC scaffolds were obtained by grafting glycidyl methacrylate (GMA) followed by amination with ethylenediamine (EDA). The amino (-NH_2_) groups on the BNC template effectively promoted the absorption of deposited CaO and SiO_2_ precursors, and thereby NBG nanofibrous scaffolds exhibited a 3D porous interconnected-network structure consisting of a 20 nm diameter nanofibre. Further, the NBG scaffold exhibited high bioactivity as measured for 7 days immersion in SBF. This study provided an insight into the 3D nanofibrous NBG scaffold for promising use in bone tissue engineering [94]. In another study, in situ calcium phosphate (CP) deposition on BNC fibrils (as sacrificial template) was processed under ultrasonication to obtain hybrid composites. It was subjected to freeze-drying (creating a 3D porous network) and then calcination treatment (removal of BNC in the 600–1200 °C range) to prepare highly crystallized 3D porous structures. Here, the heating rate and calcination time influenced the porosity and dimension of grains. As-prepared CP materials exhibited intrinsic magnetic properties that can be effective in cell attachment and growth [95]. As ideal delivery of recombinant human BMP-2 (rhBMP-2) has been a challenge in bone tissue engineering; therefore, Xiao et al. 2019 developed a mesoporous BG nanotubular (MBG-NT) scaffold by loading rhBMP-2 (184 ± 5 ng/mg) using the BNC template assisted sol-gel method (Figure 4). As-obtained 3D networked MBG-NT scaffold showed a sustained release of rhBMP-2 for 28 days due to the mesoporous architecture. In addition, rhBMP-2 loaded MBG-NT scaffold exhibited enhanced growth of human bone marrow stromal cells (hBMSCs) as compared to only MBG-NT scaffold. This scaffold system could be very promising for large bone defect regeneration [82].

Jinga et al. 2020 developed a glass-ceramic 3D spongy scaffold (CaO-BaO-P_2_O_5_/TiO_2_) by using BNC as a sacrificial template and subsequently loaded with CPs through chemical reduction and a dried gel based on barium and titanium through physical attachment. The resulted composite system was freeze-dried and then subjected to calcination treatment (>1000 °C), where the BNC network was removed completely and remained only combined mineral phases via intense diffusion. The results showed a peculiar 3D structure having porous and branched aspects with crystalline phases only and enough mechanical strength to become self-sustained. Moreover, the as-obtained scaffold exhibited no cytotoxicity to MSCs [61]. In another study, composite membranes by using BNC, CPs, and barium titanate (BaTiO_3_) were developed under ultrasonic irradiation and subjected to thermal treatment (1000–1200 °C) to obtain 3D porous structures by removing BNC as a sacrificial template. In this study, the authors investigated the effect of BaTiO_3_ to facilitate electrical stimulation to the physiological microenvironment to benefit cellular metabolism. Here, self-sustained mineral structures with high porosity (by combining non-stoichiometric phosphate phases and tetragonal BaTiO_3_) were obtained due to using BNC. The increase in calcination temperature efficiently modified the microstructure in terms of improved grain size and densification via intense diffusion. Moreover, As-obtained composite membranes exhibited biocompatibility with MSCs (adhesion and proliferation of cells by retaining elongated shape) [96].

### 5.2. Bacterial Nanocellulose (as Only Matrix)

Zang et al. 2014 investigated the efficacy of BNC and human adipose-derived stem cells (hASCs) together and showed successful osteogenic differentiation of hASCs on BNC. In addition, in vivo study confirmed the repair ability of BNC on damaged bone [97]. Further, the osteogenic potential of BMP-2-coated BNC scaffold has been investigated, where BNC/BMP-2 scaffold showed suitable biocompatibility in vitro and promoted differentiation of mouse C2C12 cells (fibroblast-like) into osteoblasts. This induced osteogenic activity could positively be correlated to the amount of BMP-2 (0~3 μg/scaffold). Moreover, the subcutaneous implantation study (in vivo) exhibited more new bone formation and higher calcium concentration in the case of BNC/BMP-2 than that of only the BNC scaffold [92]. Similarly, Koike et al. 2019 also demonstrated the efficacy of BMP-2 loaded BNC scaffold for clinical pre-dental implant in the maxillary sinus for effective alveolar bone augmentation. In this study, authors found superior properties of BNC/BMP-2 compared to only BNC or BMP-2 solution. The in vivo results of critical frontal bone defect models (male Japanese white rabbits) exhibited a sustained release of BMP-2 from BNC while maintaining graft space and accelerated new bone formation [28].

In bone tissue engineering, BMP-2 presents a therapeutic strategy clinically but required high dosages cause the challenge of cost and safety. Therefore, Dubey et al. 2021 demonstrated the efficacy of a low dose of BMP-2 through tissue engineering (by integrating 3D macro-/microporous nanofibrous BNC scaffold) or low dose BMP-2 primed murine MSCs (C3H10T1/2 cells). Unprimed cells cultured on the BNC scaffold confirmed the favorable environment of the scaffold for adhesion, growth, and infiltration due to its ECM-mimicking architecture. Further, BNC scaffold seeded with BMP-2 (50 ng/mL)-primed cells exhibited an early onset and remarkably improved bone matrix secretion and maturation compared to the unprimed scaffold. However, the BNC scaffold alone was able to ease the mineralization of cells to a limited degree. Therefore, this study provides assistance of ‘osteoconduction’ from macro-/micro-/nanofibrous architecture and ‘osteoinduction’ from a low dose of BMP-2 primed cells as a cost-effective approach for quick and outstanding osteointegration in vivo for bone tissue regeneration [98]. Due to the excellent biomaterial applicability of BNC, resorbable BNC membranes by using irradiation technique for guided bone tissue engineering (as in dental area) have been developed. In this study, this irradiation enhanced biodegradation through the cleavage of glucose bonds of BNC. The viability of NIH-3T3 cells was remarkably improved on irradiated BNC membranes (100 or 300 kGy) compared to non-irradiated BNC membranes after 3 and 7 days of culture (*p* < 0.05). Furthermore, when evaluated on rat calvarial defect models (in vivo), histometric results exhibited significantly higher new bone area (%) in the case of 100 kGy treated-BNC membranes compared to 100 kGy treated-BNC membranes after 8 weeks post-implantation (*p* < 0.05) [99]. In another study, Farnezi Bassi et al. 2020 compared the efficacy of BNC and collagen membranes in the bone regeneration of 8 mm critical size defects (rat calvaria). In this case, at 30 and 60 days post-operation, collagen membranes (positive control) exhibited efficient healing of the surgical wound with high amount of new bone formation (*p* < 0.001) than that of BNC membranes (experimental group) and control group (negative control), where BNC group showed a large amount of mature connective tissue in filling the defect. Here, higher inflammatory cell count (low biocompatibility) was observed in the BNC group than positive control at post-operative 7 and 15 days. Moreover, medium and intense immunolabeling of osteocalcin and osteopontin was observed in the immunohistochemical study at post-operative day 60 in both positive control and experimental groups. In this study, BNC membranes had no effect on bone regeneration in rat calvaria [100].

Kheiry et al. 2018 demonstrated the osteogenesis of the BNC scaffold loaded with fisetin (a phytoestrogen) as evaluated with bone marrow MSCs (BMSCs). In this study, no cytotoxicity effect of BNC/fisetin scaffold was observed on BMSCs, whereas the cell viability was enhanced. In addition, the BNC/fisetin scaffold differentiated BMSCs into the osteoblasts and exhibited the expression of osteocalcin and osteopontin genes in the cells. This study presented an effective approach to accelerate osteogenic differentiation and proper localized delivery for bone tissue engineering applications [88]. In a study, the drug release behavior of BNC-based biomaterial under physiological and antibiotic conditions was investigated to be applied in dental therapies (e.g., dental extraction or mucosal transplantation). In this study, Weyell et al. 2019 prepared oxidized-BNC to evaluate its modified degradation behavior and also doxycycline-loaded BNC to analyze their effect for the prophylaxis against infection as compared to native BNC. The obtained results confirmed the in vitro biocompatibility and antibiotic efficacy against pathogenic oral bacteria. Furthermore, a comparative biphasic release of the doxycycline was observed for native and oxidized-BNC [101].

The scaffold is composed of NFC, cyclodextrins (β-CD or methyl β-CD), and raloxifene hydrochloride (RLA, as selective estrogen receptor modulators to treat and prevent osteoporosis [102]) with suitable mechanical and osteogenic properties has been reported [103]. Therefore, bio-absorbable barrier membranes composed of BNC with β-CD for treating periodontal disease in dental medicine caused by various bacteria strains are highly desired. Here, a selectively oxidized-BNC membrane loaded with bactericide to manipulate bio-absorbing duration and a bactericide effect is a promising approach. In this case, Inoue et al. 2020 prepared o-BNC by using periodate and used chlorhexidine (CHX) as a model drug for developing bioactive membranes. In this study, inclusion complexes of CHX with β-CD were prepared to modulate the efficacy and release of CHX. This o-BNC/CHX:β-CD membrane exhibited a 10-fold enhancement in the release rate of CHX as compared to unmodified BNC. In addition, membranes loaded with CHX showed inhibition of S. aureus, E. coli, and C. albicans, but o-BNC/CHX:β-CD exhibited a larger inhibition zone (*p* < 0.05) [104].

### 5.3. Bacterial Nanocellulose/Polymer-Based Biomaterials

The scaffold can play an evaluative role in the differentiation of stem cells and thereby tissue engineering. A single material cannot provide all desired properties, and therefore, flexible composite systems are highly desired with appropriate characteristics for tissue regeneration. Here, Noh et al. 2019 developed BNC/collagen scaffolds in various ratios (1:1, 3:1, 5:1) and showed well-organized architecture with the interconnected porous network with excellent physical stability and more water-uptake capacity (up to 400%) compared to only collagen scaffold, including favorable cell adhesion and growth. Further, there were observed up-regulated osteogenic markers (e.g., collagen type 1, osteocalcin, and bone sialoprotein) and remarkably promoted proteins and calcium deposition, specifically with BNC/collagen (5:1) scaffold after osteogenic induction of umbilical cord blood-derived MSCs (UCB-MSCs) for three days. Furthermore, the subcutaneously transplanted PKH-26 pre-labeled MSCs-loaded BNC/collagen scaffolds in a mouse model exhibited several PKH-26-labeled cells and positive signals of α-smooth muscle actin for neovascularization in BNC/collagen (5:1) scaffold [85]. Klinthoopthamrong et al. 2020 prepared a non-resorbable membrane by conjugating plant-derived recombinant human osteopontin (p-rhOPN; a cost-effective RGD-containing biomolecule) for guided bone tissue regeneration. In this study, BNC was grafted with polyacrylic acid (PAA), and this BNC-g-PAA provided active anchoring sites for p-rhOPN conjugation through multiple carboxylic functional groups. The BNC/p-rhOPN membrane exhibited induced biological functions to enhance adhesion and osteogenic differentiation of human periodontal ligament stem cells (hPDLSCs) as similar to the characteristics of commercial rhOPN from mammalian cells (BNC/rhOPN) and better than BNC only [105].

### 5.4. Bacterial Nanocellulose/Filler-Based Biomaterials

**Figure 5:**
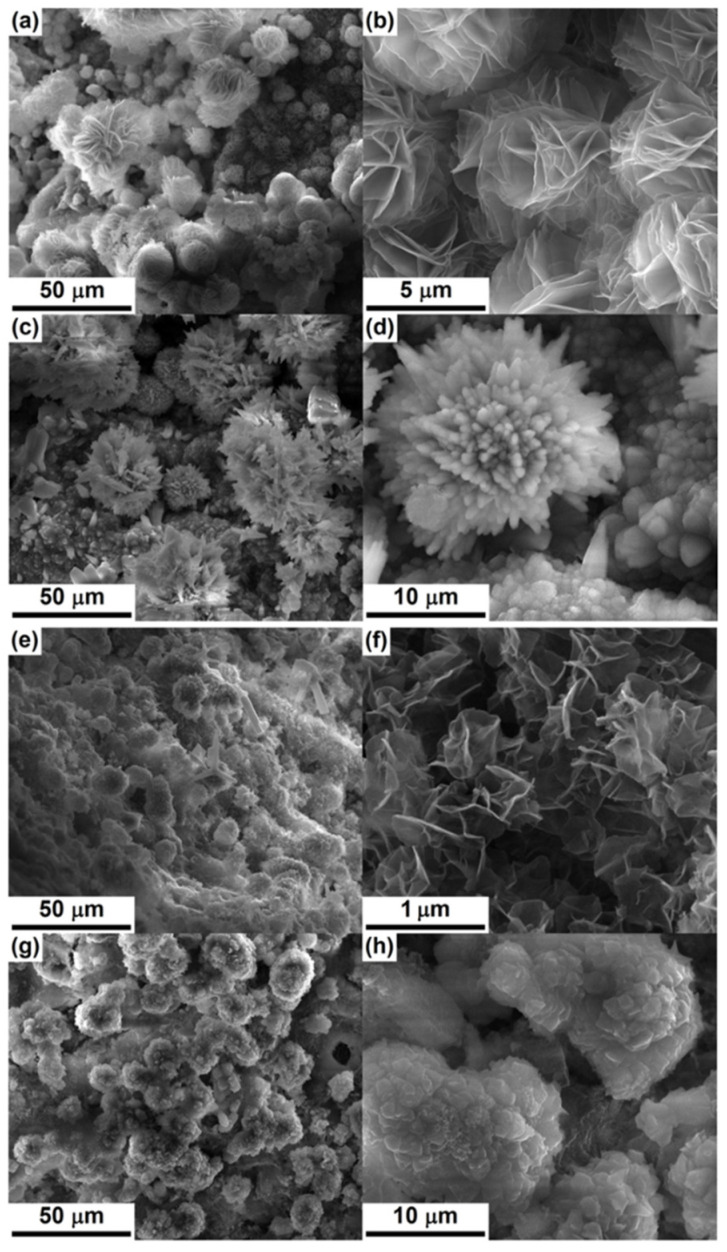
SEM images of hardened (for 28 days) unitary and cement pasts after immersion in SBF for 14 days: (**a**,**b**) C1, (**c**,**d**) C2, (**e**,**f**) C1-BNC, and (**g**,**h**) C2-BNC. Reproduced with permission from [109]. Copyright 2017 Elsevier.

Wan et al. 2011 prepared 3D nanofibrous scaffolds composed of carbon nanofibre (CaNFs) and HAp. In this study, BNC was used as starting carbon source to obtain CaNFs (diameter: 10–20 nm) by carbonization under an inert environment. In CaNF/HAp composite scaffolds, nitric-acid-treated CaNFs accelerated mineralization and modified the morphology of HAp formed onto CaNFs [106]. Costa et al. 2012 reported BNC/nano-otoliths bionanocomposite scaffold as direct dental pulp capping for suitable osteoinductive effect for bone regeneration by authorizing better cell migration to form the bone fabric. Here, otoliths as protein matrix (composed of calcium carbonate and organic phase) are found in the inner ear of the fish and are considered vital for the bone mineralization process on the nano-otoliths. As evaluated on dental pulps of dog teeth to capping, BNC/nano-otoliths scaffold promoted the formation of mineralized tissue barrier and induced reparative pulp response compared to that of the control group. This composite system exhibited better regeneration ability of the bone defect [107]. Fan et al. 2013 prepared the composite scaffolds of BNC and goat bone apatite (GBA) by a dissolving process using 4-methylmorpholine-4-oxide (NMMO) and immersing in water-bath followed by the lyophilization. The results showed homogenous dispersion of GBA into BNC matrix with reduced crystallinity and weight loss ratio of the composite scaffolds by increasing GBA content. The BNC/GBA composite scaffolds were soaked in PBS and showed a stable pH value (~7.38) of the medium (PBS) after immersing composite scaffolds for 12 weeks. Further, BNC/GBA composite scaffolds exhibited enhanced cell proliferation and accelerated cell differentiation. These results presented the promising ability of BNC/GBA scaffolds as bone filler material for the repairing of bone defects [108]. In a study, Voicu et al. 2017 prepared hybrid materials by crushing BNC in a polygranular powder through the hydrothermal process (i.e., autoclaving) followed by the mixing of it with silicate cement powders as synthesized by sol-gel method. Here, two types of thermally treated unitary cement samples were prepared at 1400 °C for 2 h as C1 and 1450 °C for 5 h as C2. BNC/silicate cement hybrid scaffold exhibited shortened setting time and a significant mineralization effect in SBF (see Figure 5). Then, cement pastes, named C1-BNC and C2-BNC, were developed for comparative analysis. Moreover, the hybrid scaffolds exhibited adhesion and proliferation of MSCs [109]. This hybrid system shows the potential to be used in dentistry.

BNC (organic phase) associated with HAp (inorganic phase) serves as potential hybrids for bone tissue regeneration due to its osteo-conductive/inductive and osteogenic characteristics. In addition, this hybrid system possesses outstanding properties, such as mechanical properties, conformability, elasticity, and cytocompatibility, owing to the synergistic behavior of both phases as compared to a single phase. In addition, the BNC/HAp multiphase system, including the incorporation of metal cations (e.g., strontium) to enhance functional ability [110]. BNC/HAp composite scaffolds are very promising in hard tissue regeneration, but it shows a few limitations due to low in vivo degradability. Therefore, to manipulate degradation behavior, Luz et al. 2020 prepared oxidized-BNC (o-BNC) by periodate-oxidation for different time periods and developed BNC/HAp or o-BNC/HAp membranes through mineralization. o-BNC/HAp composite scaffold exhibited better bioactivity and degradability than that of BNC/HAp and was dependent on the degree of BNC oxidation, and thereby, it showed a high level of glucose and other by-products (e.g., butyric acid and acidic acid) [79]. Similarly, the same research group prepared BNC or o-BNC membranes associated with HAp/strontium or strontium apatites (SrAp), such as (BNC or o-BNC)/HAp/Sr and (BNC or o-BNC)/SrAp for guided bone tissue regeneration. All composite membranes were bioactive and showed different release profiles of Sr. The oxidation of BNC improved the degradation mechanism under physiological conditions. Furthermore, composite membranes exhibited low inflammatory reactions and enhanced connective tissue repairing [80]. Sousa et al. 2020 used BNC membranes as a platform to precipitate cerium-doped calcium phosphates (Ce-CPs) by soaking BNC membranes successively in solutions having precursor ions such as Ca^2+^, PO_4_^3-^, and Ce^3+^ and then calcination treatment (600 °C for 3 h). As-obtained Ce-CPs showed three major phases as HAp, hexagonal chlorapatite, and orthorhombic buchwaldite (sodium calcium phosphate) and exhibited trabecular structure of nanowires with interconnected pores (as similar to bone tissue). The deposition of ceramic was observed on the surface of BNC fibrils. Moreover, Ce-CPs and BNC/Ce-CPs scaffold exhibited cell attachment and viability to be suitably used for bone regeneration [111]. Magnetic nanoparticles have widely been used to control cellular behavior and functions in tissue engineering applications [112]. Torgbo et al. 2019 prepared nanocomposite scaffolds composed of BNC, HAp, and magnetic nanoparticles (MNPs, Fe_3_O_4_) using ultrasonic irradiation. Here, HAp and Fe_3_O_4_ enhanced the mechanical and physiochemical properties of the BNC-HAp/Fe_3_O_4_ scaffold. This BNC-HAp/Fe_3_O_4_ scaffold showed uniform dispersion of HAp particles with Ca with a Ca/P ratio of 1.63 (surface) and 1.56 (cross-section), but decreased crystallinity of BNC in the scaffold system. In addition, BNC-HAp/Fe_3_O_4_ scaffold showed superparamagnetic property with outstanding thermal properties and improved adhesion and proliferation of human osteoblast cells (MC3T3-E1) [113].

As nanocelluloses (e.g., CNFs) have been considered very effective in fabricating BG-based composite with enhanced osteoconductivity and mechanical properties [114]. Therefore, Abdelraof et al. 2019 used BNC for preparing BNC/nano-BG-based nanocomposites using an in situ fermentation strategy. This strategy improved the yield of BNC and maintained nano-BG at the pH of the culture medium. In addition, the nanocomposite scaffold effectively enhanced the cytocompatibility and antimicrobial activities [81]. In another study, CNCs have been used together with BNC and HAp to improve the efficacy of BNC/Hap-based biomaterial. Sukyai et al. 2018 prepared BNC/HAp-CNCs (Ca/P: 1.66) composite scaffolds, where CNCs (as dispersant) were used to enhance colloidal stability of HAp during synthesis and then added into culture medium during biosynthesis of BNC. The results showed the HAp particles localized on CNCs surface and the reduction in crystallinity of CNCs (~70.90%) and HAp (~24.25%) [115]. The effect of HAp on BNC/HAp-CNCs composite scaffolds exhibited reduced crystallinity (%) and slightly enhanced thermal behavior. Moreover, composite scaffolds showed no cytotoxicity, and the cell viability was up to ~83.4% as compared to the negative control (~99.2%) [116]. In addition, carbon nanomaterials (e.g., CNTs, GO) are conductive in nature and very effective in promoting cellular behaviors. Therefore, Gutierrez-Hernandez et al. 2017 developed scaffolds composed of BNC and carboxylated-multiwalled CNTs (f-MWCNTs) and showed enhanced mechanical properties and higher adhesion, viability, and proliferation of osteoblast cells than that of traditional culture substrates [83].

### 5.5. Bacterial Nanocellulose/Polymer/Filler-Based Biomaterials

Zimmermann et al. 2011 prepared BNC/HAp nanocomposite scaffolds for bone healing applications. In this study, BNC with pellicles and tube-like surface morphologies were charged negatively by absorbing carboxymethyl cellulose (CMC) to initiate nucleation of calcium-deficient HAp (cdHAp) through the SBF immersion method. Here, the crystal size of cdHAp was enhanced with increased BNC fibril density. The Ca/P ratio was observed, ranging between 1.22 and 1.92. Moreover, scaffolds exhibited enhanced cell attachment and differentiation of osteoprogenitor cells [74]. de Olyveira et al. 2017 prepared the modified BNC by incorporating chondroitin sulfate (CTS) to the culture medium before the inoculation of bacteria (fermentation process), and as-prepared BNC/CTS was immersed in various SBF solutions to obtain BNC/CTS membranes coated with various CPs. The obtained coated membranes exhibited an influence on the wettability due to different Ca/P ratios of CPs on BNC surfaces [117]. For osteochondral defects, Kumbhar et al. 2017 prepared acellular bilayer composite scaffolds composed of BNC/HAp and BNC/glycosaminoglycans (BNC/GAG) for mimicking bone and cartilage tissues, respectively. As-obtained scaffolds exhibited suitable biocompatibility with human adipose-derived MSCs (hADMSCs), osteosarcoma cells, and human articular chondrocytes. In addition, on subcutaneous implantation, bilayered scaffolds showed outstanding in vivo biocompatibility with tissue ingrowth and without any adverse immunological reactions. Furthermore, the implanted bilayered scaffolds in osteochondral defect (generated in rat knees) caused progressive regeneration of cartilage, ECM deposition, and the subchondral bone regeneration by the host cells. Moreover, micro-CT analysis exhibited remarkably higher bone mineral density and ratio bone-to-tissue volume in the implanted bilayered scaffolds compared to the control animal group [118]. In a study, Khan et al. 2021 developed BNC/β-glucan biocomposite scaffolds reinforced with HAp and GO by using acrylic acid monomer through free-radical polymerization and freeze-drying process. In this study, the effect of various amounts of GO (0.1, 0.2, 0.3, and 0.4 g) on the properties of biocomposite scaffolds was analyzed. The results exhibited spongy microstructure with excellent stability, porosity, aqueous degradation, and mechanical properties. Here, BNC/β-glucan/HAp-GO (0.4) scaffold exhibited better antibacterial activity than that of other scaffolds formulations. In addition, this BNC/β-glucan/HAp-GO (0.4) biocomposite scaffolds showed more growth of MC3T3-E1 cells due to surface roughness, uniform interconnected pores, improved mechanical properties, and significant biochemical affinity for cell adhesion and proliferation. These characteristics of as-developed scaffolds are promising for fractured bones in the orthopedic area [84].

**Figure 6:**
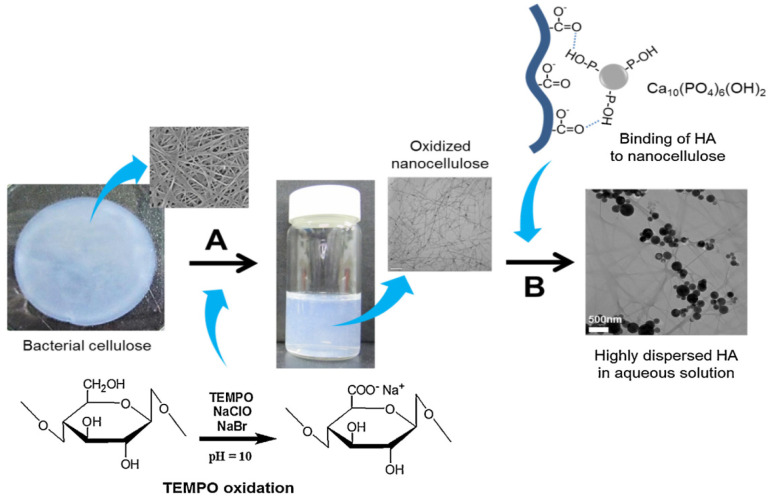
Schematic representation of TEMPO-oxidation of BNC (A) and the colloidal dispersion of HAp nanoparticles (B). Reproduced with permission from [76]. Copyright 2015 Elsevier.

Gelatin (Gel) has been used with BNC to develop BNC/Gel composite scaffolds. These composite scaffolds showed suitable penetration of Gel molecules between individual nanofibres of BNC and enhanced mechanical biological properties [75]. In addition, the oxidation of BNC influenced the properties of fabricated biomaterials. Park et al. 2015 investigated the effect of oxidized-BNC (o-BNC) as a dispersant for HAp nanoparticles in aqueous solution (Figure 6). The surface of BNC nanofibres was negatively charged after the treatment with 2,2,6,6,-tetramethylpiperidine-1-oxyl (TEMPO). This well-dispersed o-BNC-HAp colloidal solution formed hydrogel with Gel (Gel/o-BNC-HAp) by crosslinking with glutaraldehyde. The increase in Gel content enhanced the mechanical properties in both maximum tensile strength and Young’s modulus due to the increment in crosslinking of Gel and denser scaffold structure with well-dispersed o-BNC-HAp. Moreover, Gel/o-BNC-HAp scaffolds exhibited remarkable improvement in proliferation and differentiation of calvarial osteoblast cells [76].

In another study, Yang et al. 2016 also prepared o-BNC and in situ precipitated HAp/Gel system to develop nanocomposite scaffolds, where o-BNC was used as a 3D network stent. Here, both o-BNC and an increase in Gel content caused the formation of tiny HAp crystallites in the composite system. The developed o-BNC-HAp/Gel showed higher tensile strength (>0.3 MPa) and complete degradation nearly in 90 days in SBF [119]. BNC/HAp composite scaffold system provides suitable biological affinity, but its wide applicability for bone tissue regeneration is limited due to its low mechanical strength. Therefore, Ran et al. 2017 prepared a multicomponent BNC-Gel/HAp double-networked organic-inorganic composite scaffold to provide remarkable mechanical properties. BNC-Gel/HAp scaffold showed more rough surface topography and high thermal stability compared to only BNC-Gel. In addition, the mechanical strength of the BNC-Gel/HAp scaffold was greater than that of BNC/HAp or even higher than BNC/Gel. Moreover, this scaffold exhibited better adhesion and greater proliferation and differentiation of rat bone marrow-derived MSCs (rBMSCs) as compared to only BNC/Gel [120].

**Figure 7:**
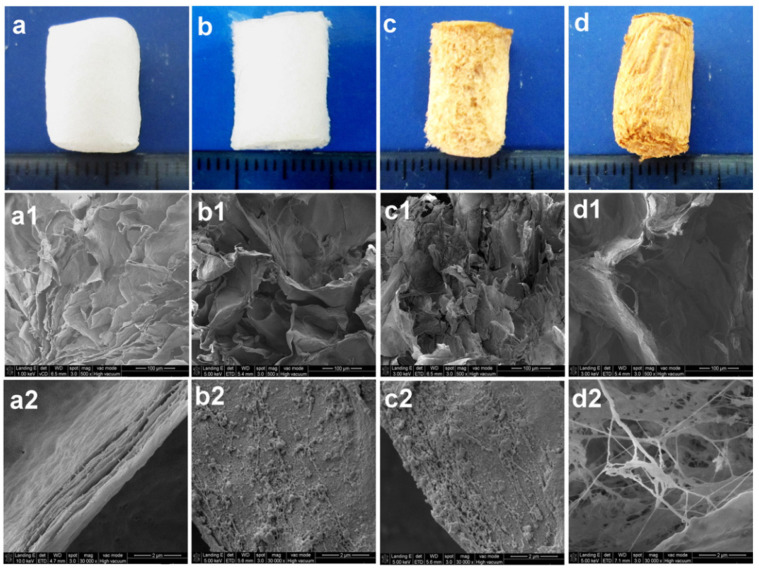
Digital images with outermost characteristics and SEM images of BNC (**a**–**a2**), BNC/Gel (**b**–**b2**), BNC-PA-Gel (**c**–**c2**), and BNC-PA-Gel/HAp (**d**–**d2**) cylindrical-shaped scaffolds. Reproduced with permission from [123]. Copyright 2017 Elsevier.

Procyanidins (PAs) are frequently used as natural antioxidants and cardiovascular protectors. Furthermore, they have demonstrated antivirus, antibacterial, anti-inflammatory, and anti-carcinogen activities. Due to their insignificant toxicity, they have been used as crosslinking agents to fix Gel without cytotoxicity [121]. Therefore, PAs have been used to crosslink BNC and Gel in BNC-Gel/HAp nanofibrous scaffolds [122]. Similarly, Huang et al. 2017 prepared an interconnected porous BNC scaffold modified with Gel through various crosslinking methods (e.g., PAs) and coated with HAp by treating with CaCl_2_ and then immersing in SBF solution (see Figure 7). The modification of the BNC scaffold showed enhanced mechanical properties as well as accelerated biocompatibility and osteoinductivity. BNC-PA-Gel/HAp scaffold showed the best adhesion, proliferation, and osteogenic differentiation of the hBMSCs. Furthermore, in vivo analyses in nude mice or rabbits exhibited higher new bone formation compared to other formulations (BNC-PA-Gel, BNC/Gel, and BNC) [123].

In addition, for mimicking bone in the scale of composition and structure, Atila et al. 2019 developed various 3D porous scaffolds composed of BNC (exopolysaccharide), HAp, or boron-doped HAp (mineral crystals), and Gel (natural protein) as a matrix by using the freeze-drying method. Comparatively, all scaffolds (Gel-BNC, Gel-BNC/HAp, and Gel-BNC/boron-doped HAp) exhibited porous structure, and pores became irregular with the incorporation of HAp or boron-doped HAp. Further, high water-uptake capacity, suitable degradation behavior, and in vitro biomineralization behavior similar to that of natural bone (Ca/P: 1.67) was observed for the scaffolds with enhanced structural stability and mechanical properties due to HAp or boron-doped HAp. Moreover, Scaffolds exhibited higher adhesion and proliferation of Saos-2 cells on Gel-BNC/boron-doped HAp and subsequently showed improved intracellular calcium deposition [124].

Alginate (Alg) hydrogels have widely been used in biomedical applications but show unstable and poor mechanical strength as well as lack of cell-recognition active sites for tissue engineering, specifically bone tissue regeneration [125,126,127]. Therefore, Yan et al. 2017 applied BNC to develop Alg/BNC-CS-Gel-based composite scaffold. In this study, hydroxyapatite-D-glucono-δ-lactone (HAp-GDL) mixture as an internal gelling system was used to prepare homogenous hydrogel, and BNC was used to improve the porous structure, intended mechanical and biological properties. In addition, layer-by-layer (LBL) electrostatic assembly of positively charged CS and negatively charged Gel was proposed to accelerate the stability and cytocompatibility of the hydrogel. The obtained scaffold showed suitable 3D microstructure with a well-defined porous network, enhanced compressive strength, and controlled biodegradation. In addition, remarkable biocompatibility and the augmented effect of BNC and external Gel chains having repetitive motifs of RGD sequences favored the adhesion, proliferation, and differentiation of MC3T3-E1 osteoblast cells [77]. Apart from other desired properties of the scaffolds, the control over complex architectural characteristics is a major concern for successful tissue regeneration. In this way, recently 3D printing technique offered a novel strategy to manufacture personalized structures for mass production in biomedical areas. Therefore, Wei et al. 2020 prepared developed a green nanocomposite printable hydrogel-ink composed of TEMPO-oxidized-BNC (o-BNC), alginate (Alg), and laponite (LAP) nanoclay. The printed o-BNC/Alg/LAP hydrogel showed structural stability (in PBS) for more than 14 days and a long-term and sustained release ability of protein. Moreover, o-BNC/Alg/LAP (<0.5%) hydrogel exhibited the ability to support cell adhesion and proliferation of L929 fibroblast cells [78]. In another study also, Aki et al. 2020 prepared a 3D printed scaffold system composed of BNC with varying amounts (0.1, 0.25, and 0.5 wt%) and the constant amounts of poly (vinyl alcohol) (PVA, 12 wt%) and hexagonal boron nitride (hBN, 0.25 wt%). As-printed BNC/PVA/hBN composite bone scaffolds exhibited homogenous dispersion of BNC within PVA/hBN matrix and showed the reduction in tensile strength with increased BNC content, whereas BNC (0.5)/PVA/hBN scaffold had the highest elongation at a break value of 93% as well as significant improvement in adhesion and viability of human osteoblast cells [73].

As reviewed and discussed in the above sections, various formulations of BNC (as a sacrificial template or only matrix) or BNC-based biomaterials for hard tissue regeneration are summarized in Table 1.

## 6. Conclusions and Future Perspective

In biomedicine, BNC can absolutely be particularly competitive in use for hard tissue replacement or restoration. Due to its versatility, in this review, we briefly discussed the synthesis and properties (including solubility, biodegradability, thermal stability, antimicrobial ability, toxicity, and cellular responses) and its surface modification approaches for understanding BNC better for future research directions. BNC has more remarkable characteristics as compared to plant-based cellulose, such as the chemically pure web-like nanofibrous network of ECM without lignin, hemicellulose, pectin, and other impurities. BNC has no animal products and possesses high crystallinity (up to 95%), long fiber length, interconnected porosity, as well as excellent mechanical properties (high wet strength), and biological properties. In addition, the in situ moldability, high water-holding capacity, high resistance to tensile deformation (in-plane) are suitable characteristics of BNC. These properties make BNC quite attractive for hard tissue engineering applications.

Pure BNC has been used as scaffolds in various studies due to its biomechanical characteristics as similar to certain biological tissues [132], but there are some major challenges, such as solution processing due to the insolubility in water and several other solvents, which limit its broader use in designing scaffolds with various materials for hard tissue engineering applications. However, this issue can be overcome by using ionic liquids for dissolution and processing through various fabrication methods. It is also very crucial to focus that the BNC forms stable hydrogel-network with very hygroscopic nature and facilitates the ultimate environment for the host cells. However, the pore size of the BNC network is not adequately large to homing the cells and thereby limits the cell penetration for further proliferation and differentiation. In addition, there is a lack of intrinsic antimicrobial activity in BNC that can be incorporated by using external antimicrobial and antibiotic agents. Furthermore, superior thermal stability prevents its melt-extrusion processability [20]. Some inherent properties (such as low porosity, non-biodegradability in vivo by enzymes, low resistance to compressive deformation in perpendicular) show limitations for commercial use of BNC. These characteristics of BNC, including others, can be manipulated by in situ or ex-situ modification approaches to enhance porosity, bioactivity, controllable biodegradation, and mechanical properties [72,133]. However, slow biodegradation of BNC-based biomaterials is beneficial for hard tissue regeneration. To extend the efficacy of the BNC, various materials (e.g., natural and/or synthetic polymers, nanomaterials, proteins, antioxidants, anti-inflammatory, antifungals, and antimicrobial agents) can be incorporated by using different design approaches (e.g., coating, gas plasma, or irradiation, chemical functionalization or other physical treatments). As a modification approach, plasma techniques are more effective in modifying the surface of BNC for optimum biofunctionalization without affecting native characteristics.

Moreover, the global pandemic situation due to COVID-19 has attracted great attention in developing materials with antimicrobial, antiviral, antioxidant properties to avoid the spreading of microorganisms and infectious diseases that threaten public health. Although extensive research reports are available on antibacterial/antimicrobial materials, very little data are available on antiviral materials. Specifically, there are huge opportunities and possibilities to develop biomaterials for tissue engineering applications (e.g., hard tissue regeneration). However, due to poor bioavailability, poor solubility, low permeability, non-targeted release, adverse side-effects, and antiviral resistance of already available antivirals, it is a little challenging to apply them extensively in potential biomedical areas [63]. However, BNC-based materials assimilated with a substance that contains significant antiviral, antifungal, anti-inflammatory, antioxidant, and antibacterial characteristics have presented great attention as biomaterials [52].

In this review, BNC or BNC-based biomaterials through different fabrication methods are reviewed and discussed to demonstrate the efficacy of BNC in hard tissue regeneration. In addition, recently additive manufacturing technologies (e.g., 3D printing or bioprinting) have also shown great potential in manufacturing 3D scaffolds with controlled and complex architectures or medical devices [134]. Here, BNC has been considered as one of the components of the hydrogel-inks/bio-inks for 3D printing/bioprinting processes [73,78,135,136,137]. However, the major challenge of using BNC in 3D bioprinting is the disentanglement of a very complex BNC fibril network formed during biosynthesis. This nanofibril system flocks and clogs the extrusion nozzle of the printer and obstructs the application of BNC as bioink [138]. Therefore, there is a need for extensive research on BNC-based 3D printed biomaterials for hard tissue regeneration. Currently, the research of BNC-based biomaterials for hard tissue regeneration is in the laboratory phase and needs extensive experimental studies from production to successful clinical translation.

Overall, despite the substantial efforts in designing economical processes for BNC production through optimizing both upstream and downstream processes, it is still challenging to enhance the scale of cultivation and is in an emerging phase for commercialization. The technological production of BNC is hugely expensive as compared to the conventional BNC fermentation methods. Economic constraints such as high capital investment, high operating costs, and low productivity of BNC are the major challenges for the commercialization and, thereby, clinical success in tissue engineering applications [19]. Therefore, the research should be focused on enhancing the production yield of BNC at a low cost. Moreover, BNC as an excellent nano-renewable biomaterial still has huge potential for biomedical applications.

## Figures and Tables

**Table 1:** Potential BNC-based biomaterials for dental and bone tissue regeneration.

Composition	Scaffold Form	Cell/Drug/Biomolecule	Features	Ref.
BNC	Membrane	NIH-3T3 fibroblast cells	Suitable biocompatibility and enhanced cell viability, remarkably formation of large new bone area	[99]
BNC	Membrane		Low biocompatibility and large amount of mature connective tissue in filling the defect (adult male rat)	[100]
BNC	Nanofibrous	BMP-2, C2C12 cells	Suitable biocompatibility and osteogenic differentiation of fibroblast-like cells, and BNC scaffold with BMP-2 exhibited more bone formation and higher calcium content than that of BNC only	[92]
BNC	Micro-/nanofibrous	Osteoblasts and fibroblasts, BMP-2	Promoted optimal bone formation and sustained release of BMP-2	[28]
BNC	Macro-/micro-/nanofibrous	C3H10T1/2 cells, BMP-2	Low dose of BMP-2 exhibited excellent cell adhesion and growth, remarkably improved bone matrix secretion and maturation, and facilitated the mineralization of cells to some extent	[98]
BNC	Nanofibrous	HASCs	Successful osteogenic differentiation of HASCs on BNC and tissue-repairing ability	[97]
BNC	Nanofibrous	L929 fibroblasts, doxycycline	Suitable biocompatibility and antibiotic efficiency against pathogenic oral bacteria	[101]
BNC/β-CD-CHX	Membrane	CHX	Ten-fold increase in release rate of CHX, all CHX-loaded membranes showed antibacterial activity, but BNC/β-CD-CHX exhibited greater inhibition zone	[104]
BNC/collagen	Fibrous	UCB-MSCs and NIH3T3 cells, BMP-2, dexamethasone	Favorable cell adhesion and growth, more up-regulated osteogenic markers and remarkably uplifted proteins and calcium deposition, and positive signals (α-smooth muscle actin) for neovascularization	[85]
BNC/collagen	3D mesoporous microspheres	MC3T3-E1 cellsBMP-2	High surface area, suitable biocompatibility, effective promotion of cell adhesion, proliferation, and osteogenic differentiation	[128]
BNC/Gel	Nanofibrous	NIH-3T3 fibroblast cells	Decreased crystallinity and improved thermal stability, Enhanced Young’s modulus and decreased tensile strength, and excellent biocompatibility	[75]
BNC/MWCNTs	Nanofibrous	Osteoblastic cells (human inferior maxillary bone)	Excellent adhesion and proliferation of osteoblastic cells	[83]
BNC/fisetin	Nanofibrous	BMSCs	Suitable cytocompatibility with enhanced cell viability, differentiation of BMSCs to osteoblasts and promoted the expression of osteocalcin and osteopontin genes	[88]
BNC/otoliths			Stimulation of the formation of mineralized tissue barrier and reparative pulp reaction	[107]
BNC/goat bone apatite	3D porous	L929 fibroblasts	Suitable bioactivity and stimulation of cell proliferation and differentiation	[108]
BNC/HAp	Nanofibrous		3D porous network with homogenous precipitation of carbonated-HAp crystals on BC fibers	[129]
BNC/HAp	Nanofibrous		3D porous network with homogenous precipitation of carbonated-HAp crystals on BC fibers	[130]
BNC/HAp	Nanofibrous		Oxidized-BNC/HAp is more bioactive and degradable than BNC/HAp and high glucose levels in BNC degradation	[79]
BNC/HAp	Nanofibrous		Surface-treated carbon nanofibres (CNFs) (from BNC) showed enhanced biomineralization and changed morphology from needle-like to rod-like HAp formed on CNFs	[106]
BNC/HAp-CNCs	Nanofibrous		CNCs-assisted dispersibility of HAp exhibited promising results	[115]
BNC/HAp-CNCs	Nanofibrous	L929 fibroblasts	Suitable dispersibility and had less effect of HAp/CNCs on crystallinity, whereas slight increase in thermal stability, and suitable cytocompatibility	[116]
BNC/MNPs/HAp	Nanofibrous	MC3T3-E1 cells	Enhanced mechanical and physiochemical properties, superparamagnetic and remarkable thermal stability, and significant cell adhesion and proliferation	[113]
BNC/HAp/Sr and BNC/SrAp	Porous membrane	L929 fibroblasts	Oxidized-BNC/SrAp exhibited improved degradation under physiological conditions with suitable cytocompatibility, low inflammatory reaction, and enhanced connective tissue repair, including degradation (in vivo)	[80]
BNC/HAp/Sr and BNC/SrAp	Porous membrane	L929 fibroblasts	Oxidized-BNC/SrAp exhibited improved degradation under physiological conditions with suitable cytocompatibility, low inflammatory reaction, and enhanced connective tissue repair, including degradation (in vivo)	[80]
BNC-PVP/HAp (in situ using SBF)	Nanofibrous		Improved apatite formation ability of BNC with higher HAp deposition	[131]
BNC-PA-Gel/HAp	Nanofibrous	MSCs	Excellent cellular compatibility and bone-like properties	[122]
BNC-PA-Gel/HAp	Fibrous structure	hBMSCs and rBMSCs	Excellent mechanical properties and cytocompatiblity (adhesion, proliferation, and osteogenic differentiation), and high new bone formation	[123]
BNC-HAp/BC-GAG	Bilayer	Osteosarcoma cells, hADMSCs, and human articular chondrocytes	Suitable tissue ingrowth and no adverse immunological responses, progressive regeneration of cartilage tissue, ECM deposition, and subchondral bone regeneration, and remarkably higher mineral density and volume ratio of bone to tissue	[118]
BNC-Gel/HAp	Nanofibrous		Oxidation of BNC and increased content of Gel induced the formation of tiny HAp crystallites and Gel (0.3 wt%)-incorporated composite system exhibited promising effects	[119]
BNC-Gel/HAp	Nanofibrous	Calvarial osteoblasts	Excellent mechanical properties and improved cell proliferation and differentiation	[76]
BNC-Gel/HAp	Nanofibrous	rBMSCs	Rough surface morphology, enhanced mechanical properties, better adhesion, and higher proliferation and differentiation of cells	[120]
BNC-boron-doped HAp/Gel	3D porous	Saos-2 cells	Suitable degradation rate and in vitro bioactivity, excellent cytocompatibility, and intracellular calcium deposition	[124]
BNC-CMC/HAp (in situ using SBF)	Nanofibrous	Osteoprogenitor cells (MC3T3-E1)	Calcium-deficient HAp enhanced BNC fibril density and improved cell attachment and growth	[74]
BNC/Alg-CS-Gel/HAp		MC3T3-E1 cells, RGD	Suitable 3D structure with well-defined porous network, enhanced compressive properties, and remarkable biocompatibility	[77]
BNC-β-glucan/HAp-GO	3D porous	MC3T3-E1 cells	Suitable mechanical and antibacterial properties, significant cell adhesion and proliferation	[84]
BNC/CPs	Nanofibrous	AFSCs	BNC was used as template and calcinated to prepare 3D calcium phosphate-based scaffold as bioactive filler or bone tissue regeneration with suitable biocompatibility and bioactivity	[93]
BNC/CPs	Membrane	CHO-K1 cells	Suitable deposition of calcium phosphate and wettability, and suitable cytocompatibility	[117]
BNC/CPs	3D fibrous		Suitable intrinsic magnetic properties for effective cell adhesion and growth	[95]
BNC/cerium-doped-CPs	Nanofibrous	GM07492 human fibroblasts	Achieved trabecular morphology with interconnected pores and suitable cell viability	[111]
BNC/CPs/barium titanate (CaO-BaO-P_2_O_5_/TiO_2_)	3D porous	hMSCs	Only crystalline phase emerged as TiO_2_ in 3D structure and exhibited no cytotoxic effect	[61]
BNC/CPs/BaTiO_3_	3D fibrous	MSCs	BNC-acted as sacrificial template and scaffold exhibited suitable biocompatibility	[96]
BNC/BG		Vero cells	Improved BNC yield with enhanced biocompatibility and antimicrobial properties	[81]
BNC/BG	Nanofibrous		BNC was used as template and calcinated to prepare highly bioactive 3D nanofibrous BG-based scaffold with high bioactivity	[93]
BNC/BG	Nanofibrous		Eeffective absorption of deposited CaO and SiO_2_ precursors on the surface of BNC, 3D porous interconnected-NBG nanofibrous scaffolds, and higher bioactivity	[94]
BNC/mesoporous BG	Nanofibrous	hBMSCs, rhBMP-2	A sustained release of rhBMP-2 for 28 days and enhanced cell proliferation and osteogenic-related gene expression	[82]
BNC/silicate glass	3D structure	MSCs	The behavior of BNC with silicate glasses (cements) exhibited enhanced features, especially in terms of setting time (i.e., faster) and biological properties as cell survival and accelerated cell proliferation	[120]
BNC-PVA/hexagonal boron nitride	Microporous (printed)	human osteoblast cells	Well-defined porous structure with significantly enhanced cell viability and mechanical properties	[73]
BNC-Alg/LAP	Microporous (printed)	L929 fibroblast cells, BSA	Excellent printability, improved stability of printed hydrogel with sustained and long-term protein delivery due to nanoclay	[78]
